# Medical Information Mart for Intensive Care: A Foundation for the Fusion of Artificial Intelligence and Real-World Data

**DOI:** 10.3389/frai.2021.691626

**Published:** 2021-05-31

**Authors:** Paul Rogers, Dong Wang, Zhiyuan Lu

**Affiliations:** Division of Bioinformatics and Biostatistics, FDA National Center for Toxicological Research, Jefferson, AR, United States

**Keywords:** MIMIC, artificial intelligence, machine learning, ICU, critical care, database

## Introduction and Background

The Medical Information Mart for Intensive Care (MIMIC) is a database of de-identified electronic health records (EHR) associated with patients who stayed in intensive care units (ICU) at the Beth Israel Deaconess Medical Center in Boston, MA (Johnson et al., 2016). Currently on its fourth iteration, the database is maintained by the Massachusetts Institute of Technology’s Laboratory for Computational Physiology. With time frames and release dates shown in Table 1, all four MIMIC versions are accessible and available to the public, supporting the concept of reproducibility within ICU research (Johnson et al., 2018). Among these, MIMIC-III alone contains 53,423 distinct adult hospital admissions to one of the five different ICU departments at the Beth Israel Deaconess Medical Center between 2001 and 2012 (Johnson et al., 2016). These specialized ICU departments include Coronary Care, Cardiac Surgery Recovery, Medical Intensive Care, Surgical Intensive Care, and Trauma Surgical Intensive Care units. MIMIC is populated with data from hospital electronic health records, automated critical care information systems, and the Social Security Administration Death Master File. The two critical care information systems which provided time-stamped physiological measurements and progress notes were the Philips CareVue Clinical Information System and iMDsoft MetaVision ICU. Classes of MIMIC data include patient demographics, billing information, unstructured text (notes), prescription medications, vital signs, laboratory results, and a plethora of physiological measurements.

**TABLE 1:** Versions of MIMIC.

Version	Years Spanned	Release Date
MIMIC	1994–1996	March 2000
MIMIC-II	2001–2007	February 2010
MIMIC-III	2001–2012	September 2016
MIMIC-IV^a^	2008–2019	August 2020

a MIMIC-IV was released as an extension to MIMIC-III.

Due to the granularity of information, MIMIC serves as a real-world data (RWD) foundation for artificial intelligence (AI) and machine learning (ML) research applications. MIMIC places thousands of ICU records with millions of physiological measurements directly into scientists' hands, which serves as a springboard for numerous projects in the development of AI/ML algorithms to support the work of ICU clinicians and staff. Here, we highlight a few of MIMIC’s contributions within the world of AI and ML, referring to this fusion as Artificial Intelligence for Real-World Data (AI4RWD). For the sake of brevity, a limited number of these efforts are mentioned in this short overview.

## MIMIC Facilitates AI/ML Research With RWD

As the largest publicly available EHR ICU dataset, MIMIC has received intense interest from the AI/ML community as a common ground for developing and validating new methods. For example, in terms of ICU sepsis treatment, MIMIC has been used in various AI/ML approaches ranging from gradient-boosting to reinforcement and deep learning (Komorowski et al., 2018; Dauvin et al., 2019; Kaji et al., 2019; Hou et al., 2020). Several projects have focused on predicting ICU mortality, producing results that include a decision support tool for determining optimal patient ICU discharge times to improve patient outcomes (McWilliams et al., 2019). There are ICU vital signs monitoring programs, based on fuzzy models, that perform with a high degree of accuracy (Leite et al., 2011). These mentioned studies are a sample out of hundreds that employ MIMIC data to explore and develop AI applications for the critical care environment.

Prediction is the goal of most ML tasks that use the MIMIC EHRs. Generally, approaches to prediction involve the application of statistics, ML, and AI to arrive at post-admission outcomes. Logistic regression, proportional hazards model, random forests, support vector machines, boosting, and neural networks are some of the more common statistical and AI/ML approaches to these problems of prediction (Nemati et al., 2018; Vincent et al., 2018; Lin et al., 2019; McWilliams et al., 2019; Le et al., 2020; Nallabasannagari et al., 2020). These methods have been used extensively in other areas for regression or classification tasks depending on the nature of the response variable (continuous, categorical, time-to-event). The advantages and disadvantages of these methods have been extensively discussed. However, EHR data’s complex and heterogeneous nature can make directly transplanting approaches from other domains difficult. Recent developments in deep neural networks and natural language processing (NLP) have further expanded the scope of these applications (Shickel et al., 2017).

Like other EHR datasets, one significant challenge of working with MIMIC data is the high degree of inherent medical heterogeneity (MIT Critical Data, 2016). In contrast to the more homogeneous input tasks like image processing, MIMIC data spans free text forms (clinical notes, radiological reports), medical codes (ICD9/10), demographic information, time-series for vital signs, laboratory results, and medication records. Extensive data preprocessing is needed for any modeling effort that utilizes this data because feature engineering requirements are significant to the derivation of meaningful outcomes. Deep neural network-based NLP approaches, in turn, provide powerful tools to incorporate information from free-text clinical notes (Alsentzer et al., 2019). These challenges make deep learning development, with its capacity for hierarchical feature construction and long-range dependency modeling, valuable for analyzing EHR data (Shickel et al., 2017).

By applying deep learning and NLP approaches to MIMIC data, notable advances have been made in concept and patient representation and concept extraction in addition to outcome prediction (Beaulieu-Jones et al., 2018; Alsentzer et al., 2019; Huang et al., 2019; Nuthakki et al., 2019; Killian et al., 2020; Magna et al., 2020). The capacity for representation learning is significant because it enables common downstream tasks such as regression or classification. It also opens up the possibility for applications like computational phenotyping and clinical data de-identification. Going forward, MIMIC is expected to continue to be a valuable resource for the development and validation of ML/AI methods with applications to EHR data. One potential area of development that will benefit from MIMIC is causal inference, especially in conjunction with propensity score modeling and targeted maximum likelihood estimation. This holds the promise of harnessing the vast predictive power of AI/ML in the pre-final estimation steps while maintaining the well-established causal modeling framework (Blakely et al., 2021).

## MIMIC Serves as a Conduit for Collaboration

Critics of AI and ML methods developed on MIMIC raise concerns about lack of validation in terms of safety and failure to demonstrate real-world effectiveness in the clinical setting. They cite that AI acceptance will be limited without stakeholder engagement and will suffer from a limited understanding of the critical care environment (Herasevich et al., 2020). These criticisms are not without merit, but they fail to recognize that these initial studies establish a basis for implementing an AI- or ML-based medical application designed to support the ICU clinician. Publicly available critical care datasets like MIMIC provide a collaborative “sandbox” that brings together clinicians, academia, medical technology companies, and the pharmaceutical industry. The public availability of MIMIC, combined with the granularity and volume of ICU data, fabricates a “crucible” where data scientists’ and medical practitioners’ ideas and efforts are “forged” together. This merging of disciplines is manifested at international datathons centered around MIMIC including the United Kingdom, China, Australia, Brazil and Singapore. Currently, MIMIC is studied in over 30 countries, with 4,000-plus users in industry and academia (Pollard and Celi, 2017). These alliances create unlimited potential for AI applications within the ICU, ultimately promoting positive patient outcomes and easing clinician workloads.

## Utility in Regulatory Science

AI systems that assist in decision-making for individual patients are referred to as clinical AI by the National Academy of Medicine (Matheny et al., 2019). The performance of these systems undergoes intense scrutiny by the U.S. Food and Drug Administration (FDA) and its international counterparts. The challenges faced by these regulatory bodies include the collection of dynamic data (i.e., wearable devices) combined with RWD, such as EHRs; providing effective oversight and validating these systems requires more than a static, limited dataset. Because clinical AI systems are deployed to different institutions servicing different communities, epidemiological drift will likely require some fine-tuning of the algorithms to incorporate data from diverse populations (Matheny et al., 2019). In conjunction with other dynamic ICU EHRs, MIMIC can serve as one building block for calibrating these algorithms over time.

The FDA states that “Artificial intelligence and machine learning technologies have the potential to transform health care by deriving new and important insights from the vast amount of data generated during the delivery of health care every day” (FDA, 2021). In other words, regulatory oversight of clinical AI systems is essential to ensure safety, quality, and most importantly, public trust. MIMIC is a treasure trove of ICU data collected during the daily delivery of critical care to patients. As such, the insights provided by the AI/ML project initiatives, based upon MIMIC, serve as additional support for decision-making within regulatory science. An example of the way MIMIC facilitates the regulatory mission involves the antidiarrheal medication Loperamide. In 2016 the FDA issued a safety communication regarding serious heart problems associated with the use of Loperamide (FDA, 2016). There are 2,309 prescriptions of Loperamide captured within MIMIC-III, allowing further study and modeling of the drug relative to patient demographics, vital signs, and other factors of interest in the critical care environment (Adibuzzaman et al., 2016). Thus, the findings derived from MIMIC can offer opportunities to advance public health by improving and managing critical care outcomes.

## Conclusion

MIMIC is an ICU real-world data set that serves as a research catalyst fusing the AI and ML efforts of technologists, academic researchers, regulators, and clinicians together to improve the critical care environment. It is foreseeable that MIMIC, in conjunction with other ICU datasets, may provide regulatory agencies with a bird’s-eye view of the post-market performance of new critical-care AI devices and systems. MIMIC serves as a cornerstone that brings AI and RWD together into an innovative mix, spawning ideas, algorithms and laying the groundwork for new critical care medical technologies.
